# Antioxidant and Antisenescence Effects of Bergamot Juice

**DOI:** 10.1155/2018/9395804

**Published:** 2018-07-12

**Authors:** Eleonora Da Pozzo, Marinella De Leo, Immacolata Faraone, Luigi Milella, Chiara Cavallini, Eugenia Piragine, Lara Testai, Vincenzo Calderone, Luisa Pistelli, Alessandra Braca, Claudia Martini

**Affiliations:** ^1^Department of Pharmacy, University of Pisa, Via Bonanno 6, 56126 Pisa, Italy; ^2^Research Centre for Nutraceutical and Healthy Foods “Nutrafood”, University of Pisa, Via del Borghetto 80, 56124 Pisa, Italy; ^3^Department of Science, University of Basilicata, Viale dell'Ateneo Lucano 10, 85100 Potenza, Italy

## Abstract

Aging is one of the main risk factor for the onset of cardiovascular diseases; one of the possible explanations could be linked to the age-associated overproduction of free radicals. This increase of oxidative stress can be overcome with a high intake of food antioxidants. In this context, a number of studies have been addressed to assess the antiaging potential of natural antioxidant compounds. Recently, it has been shown that the juice of bergamot (*Citrus bergamia* Risso et Poiteau), a fruit mostly produced in the Ionian coastal areas of Southern Italy (Calabria), is a valuable source of health-promoting constituents with, among other, antioxidant properties. In order to investigate the potential antiaging effects of this Mediterranean natural antioxidant source, bergamot juices of three different cultivars (“fantastico,” “femminello,” and “castagnaro”) were herein characterized by the mean of high-performance liquid chromatography-photodiode array-electrospray ionization-tandem mass spectrometry. Then, juices were investigated for the evaluation of total polyphenolic and flavonoid contents, cell-free model antioxidant activities, and *in vitro* antiaging properties on two different cellular models of induced myocardial senescence. The best performing juice was also assessed *in vivo*. The phytochemical profiles confirmed that juices were rich in flavonoids, both flavone and flavanone glycosides. In addition, two limonoid glycosides were also identified in all cultivars. Each cultivar showed different phenolic and flavonoid contents. In tube results showed the juice robust antioxidant activities that correlate with their phenolic and flavonoid contents. Moreover, for the first time, the ability of juice to counteract the chemical-induced senescence was here demonstrated in both cellular models. Lastly, the *in vivo* data obtained from mouse hearts evidenced an increase in transcription of genes involved in antiaging and antioxidant responses. The overall results suggest that bergamot juice exerts antioxidant and antisenescence effects, making it useful for nutraceutical purposes.

## 1. Introduction

The pathogenesis of many age-associated diseases can be due to an increase of oxidative stress; indeed, the overproduction of free radicals could impair protein and fatty acid functions and could lead to DNA damage, predisposing to age-related disorders [1]. In particular, old age is a primary nonmodifiable risk factor for the onset of cardiovascular diseases. DNA damage and telomere shortening are associated with cellular senescence, atherosclerosis, coronary artery disease, and heart failure [2]. Furthermore, the accumulation of dysfunctional mitochondria in aged cardiomyocytes could alter the redox state and contribute to the pathological myocardial remodeling and heart failure [3].

In this line, a great deal of interest has been recently devoted to the antiaging potential of natural compounds with antioxidant properties [4]. Epidemiological studies have evidenced that a high intake of antioxidant-containing food is associated with a reduced risk of cardiovascular diseases. In particular, the Mediterranean diet (containing antioxidants, omega-3 and -6 polyunsaturated acids, polyphenols, flavonoids, and procyanidins) is associated with higher longevity and reduced risk of age-related vascular disease development [5]. A number of human dietary intervention trials have shown improvements in vascular function following the consumption of foods with high flavonoid content [6, 7]. Among these foods, *Citrus* fruits have gained a high attention for their content of flavonoids and other beneficial components. In particular, bergamot (*Citrus bergamia* Risso et Poiteau), a fruit mostly produced in the Ionian coastal areas of Southern Italy (Calabria), has been demonstrated to be a valuable source of health-promoting molecules that contribute to its antioxidant, anti-inflammatory, and cholesterol reduction capacities [8–17]. *C. bergamia* is a small tree; it belongs to the Rutaceae family, mainly cultivated in a specific area of Calabria, characterized by a microclimate suitable for its growth. Whereas the bergamot essential oil obtained from the fruit peel is extensively characterized [18–20] and used in cosmetic and food industries, only recently the bergamot by-products, such as the pulp and its juice, were evaluated for their healthy biological properties [8, 11, 21–25]. This reevaluation could represent an economic advantage in the industrial processes, reducing costs of their disposal and gaining a good source of beneficial compounds. Furthermore, the promotion of the bergamot fruit may have important economic implications for the regions in which this fruit can be cultivated.

To the best of our knowledge, no studies investigating antisenescence property of bergamot juice have been performed so far. Thus, in order to explore the potential cardiovascular antiaging effects of this natural antioxidant source, bergamot juices of three different cultivars (“fantastico,” “femminello,” and “castagnaro,” usually used in the industry) were herein characterized by the means of high-performance liquid chromatography-photodiode array-electrospray ionization-tandem mass spectrometry (HPLC-PDA-ESI-MS/MS). Then, the juices were investigated for the evaluation of total polyphenolic (TPC) and flavonoid (TFC) contents and for the cell-free model antioxidant activities by superoxide radical scavenging activity (O_2_^•−^), ferric reducing antioxidant power (FRAP), and lipid peroxidation inhibition (*β*-carotene bleaching, BCB) tests; finally, the relative antioxidant capacity index (RACI) was calculated. *In vitro* antiaging properties were tested on two different cellular models of chemical-induced senescence of myocardial cell line H9c2 derived from rat hearts. Lastly, the best performing juice was also assessed *in vivo*.

## 2. Materials and Methods

### 2.1. Chemicals and Apparatus

Solvents such as methanol, chloroform, phosphoric acid, hydrochloric acid, and glacial acetic acid were purchased from Carlo Erba (Milano, Italy). *N*,*N*-dimethylformamide (DMF) was obtained from Alfa Aesar (Karlsruhe, Germany). Acetonitrile HPLC grade were purchased from VWR (Milano, Italy). HPLC grade water (18 mΩ) was prepared by a Mill-Q purification system (Millipore Corp., Bedford, MA, USA). Dimethyl sulfoxide (DMSO), doxorubicin (DOX), hydrogen peroxide (H_2_O_2_), *p*-formaldehyde, Folin-Ciocalteu reagent 2 N, sodium carbonate, aluminum chloride, sodium nitrate, sodium nitroprusside (SNP), sulfanilamide, N-(1-naphthyl) ethylenediamine dihydrochloride, phenazine methosulphate (PMS), *β*-nicotinamide adenine dinucleotide (NADH), nitrotetrazolium blue chloride (NBT), potassium phosphate monobasic (KH_2_PO_4_), sodium acetate trihydrate, 2,4,6-tripyridyl-s-triazine (TPTZ), iron(III) chloride (FeCl_3_·6H_2_O), *β*-carotene, linoleic acid, Tween 20, sodium hydroxide, gallic acid, quercetin, naringin, ascorbic acid, 6-hydroxy-2,5,7,8-tetramethylchroman-2-carboxylic acid (Trolox), butylhydroxytoluen (BHT, 2,6-bis (1,1-dimethylethyl)-4-methylphenol), Dulbecco's modified Eagle's medium (DMEM), fetal bovine serum (FBS), penicillin, and streptomycin were purchased from Sigma-Aldrich (Milano, Italy). CellTiter 96® AQ_ueous_ Non-Radioactive Cell Proliferation Assay (MTS) was obtained from Promega Italia Srl (Milano, Italy).

All spectrophotometric measurements were done in cuvettes or 96-well microplates on a UV/vis spectrophotometer (SPECTROstar Nano, BMG LABTECH).

### 2.2. Plant Material

Three different cultivars of *C. bergamia* (“fantastico,” “femminello,” and “castagnaro”) were supplied by Young Fruit and Visalli Diego companies (Reggio Calabria, Italy). Fresh fruits of each cultivar were squeezed using a manual citrus juicer, and the obtained juices were stored at −20°C and defrosted to room temperature before analyses.

### 2.3. Sample Preparation

For HPLC-PDA-ESI-MS study, 10.0 mL of each juice was added to DMF (10.0 mL) and the mixture was centrifuged three times for 5 min at 1145 ×g. The supernatant liquid was filtered through a 3 mm diameter PTFE membrane with a 0.45 *μ*m pore size. Aliquots of 10 *μ*L were injected into the LC-MS system.

For the evaluation of TPC, TFC, and antioxidant activities, juices were lyophilized and then dissolved in methanol. For the assessment of antisenescence activity, lyophilized juices were dissolved in DMSO. The samples were sonicated and centrifuged.

### 2.4. HPLC-PDA-ESI-MS/MS Analyses

Qualitative HPLC-PDA-ESI-MS/MS analyses were performed using a Surveyor LC pump, a Surveyor autosampler, coupled with a Surveyor PDA detector, and a LCQ Advantage ion trap mass spectrometer (Thermo Finnigan) equipped with Xcalibur 3.1 software. Analysis was performed using a 4.6 × 150 mm, 4 *μ*m, Synergi Fusion-RP column (Phenomenex). The eluent was a mixture of acetonitrile (solvent A) and water (solvent B). The solvent gradient was as follows: 0–15 min 5–20%, 15–40 min 20–70% A, and 40–45 min 70–100% A. Elution was performed at a flow rate of 0.8 mL/min with a splitting system of 2 : 8 to the MS detector (160 *μ*L/min) and PDA detector (640 *μ*L/min), respectively. The volume of the injected juice DMF solutions was 10 *μ*L. Analyses were performed with an ESI interface in the negative mode. The ionization conditions were optimized, and the parameters used were as follows: capillary temperature, 210°C; capillary voltage, −10.0 V; tube lens offset, −50.0 V; sheath gas flow rate, 60.00 arbitrary units; auxiliary gas flow rate, 20.00 arbitrary units; spray voltage, 4.50 kV; and scan range of *m*/*z* 150–1200. In the MS/MS experiments, normalized collision energy 35.0% was applied. N_2_ was used as the sheath and auxiliary gas. PDA data were recorded with 200–600 nm range with two preferential channels as the detection wavelength 280 and 325 nm. In order to determine the amount of main bergamot juice flavonoids, calibration curve was constructed by using the naringin as external standard in a concentration range 0.0020–0.25 mg/mL and DMF as solvent. Six different concentrations of standard solutions (0.250, 0.125, 0.060, 0.030, 0.0075, and 0.002 mg/mL) were prepared and analysed by triplicate injections. The calibration curve was generated by using concentration (mg/mL) with respect to the area obtained from the integration of the PDA/UV peaks recorded at 325 nm. The relation between variables was analyzed using linear simple correlation. For the linear regression of the standard, *R*^2^ was 0.9997. The flavonoid amounts were obtained by using a GraphPad Software Prism 6.0 and finally expressed as mg/mL of fresh juice.

### 2.5. Total Polyphenol Content

Folin-Ciocalteu assay was used to determine the total polyphenol content of samples [26]. Distilled water (425 *μ*L) and 75 *μ*L of the diluted sample (methanol in blank) were added to 500 *μ*L of Folin-Ciocalteu reagent and 500 *μ*L of Na_2_CO_3_ (10% *w*/*v*). The mixture was mixed and incubated for 1 h in the dark at room temperature. The absorbance was measured at 723 nm after incubation. Gallic acid was used as standard, and results were expressed as mg gallic acid equivalents per gram of the dried sample (mg GAE/g) from three independent assays.

### 2.6. Total Flavonoid Content

One hundred *μ*L of each sample (methanol in blank) was added to 15 *μ*L of 5% NaNO_3_ into the microcentrifuge tube. After 5 min, 30 *μ*L of 10% AlCl_3_ was added; after some minutes, 100 *μ*L of 1 M NaOH solution and 255 *μ*L of distilled water were added. The absorbance was measured against blank at 510 nm after 10 min of incubation at room temperature. Quercetin was used as standard, and the total flavonoid content was expressed as mg of quercetin equivalents per gram of the dried sample (mg QE/g) from three independent assays [27].

### 2.7. In Tube Activity

#### 2.7.1. Superoxide Radical (O_2_^•−^) Scavenging Activity

Superoxide radicals (O_2_^•−^) were generated in the PMS/NADH system, as previously reported [28]. The reaction mixture consisted of 40 *μ*L of different concentrations of the sample (methanol in negative control), 40 *μ*L of NADH, and 130 *μ*L of NBT. The reaction was started by adding of PMS (40 *μ*L) to the mixture. The superoxide scavenging capacity of methanol samples was quantified by their ability to inhibit NBT reduction to blue formazan by superoxide. The assay was conducted at room temperature, and the absorbance of formazan produced was determined at 560 nm for 2 min in kinetic function. Results were expressed in mg/mL as concentration of the sample required to inhibit the activity by 50% (IC_50_) from three independent assays. Ascorbic acid was used as positive control.

#### 2.7.2. Ferric Reducing Antioxidant Power

The ferric reducing antioxidant power of samples was determined using FRAP assay with some modifications [29]. Briefly, 25 *μ*L of appropriately diluted sample (methanol in blank) was added to 225 *μ*L of FRAP reagent and incubated at 37°C for 40 min in the dark. FRAP reagent was prepared fresh before experiment by mixing 300 mM acetate buffer in distilled water pH 3.6, 20 mM FeCl_3_·6H_2_O in distilled water, and 10 mM TPTZ in 40 mM HCl in a proportion of 10 : 1 : 1. The reduction of a colorless ferric complex (Fe^3+^-tripyridyltriazine) to a blue-colored ferrous complex (Fe^2+^-tripyridyltriazine) by action of electron-donating antioxidants was determined at 593 nm. Trolox was used as standard, and FRAP values were expressed as mg of Trolox equivalents per gram of the dried sample (mg TE/g) from three independent assays.

#### 2.7.3. Lipid Peroxidation Inhibition Assay

The ability of samples to prevent the inhibition of lipid peroxidation was carried out by BCB assay as reported by Dekdouk et al. [26]. A stock solution of *β*-carotene/linoleic acid was made by dissolving 0.2 mg of *β*-carotene in 0.2 mL of chloroform, linoleic acid (20 mg), and Tween 20 (200 mg). The chloroform was completely removed by a rotary evaporator, and distilled water (50 mL) was added with oxygen. The resulting emulsion was vigorously stirred. Aliquots (950 *μ*L) of the mixture were transferred to test tubes containing 50 *μ*L of the sample (the final concentration for all tested samples was 0.25 mg/mL) or methanol as blank. BHT was used as a positive standard. 250 *μ*L of this solution was transferred to a 96-well plate, and outer wells were filled with 250 *μ*L of water to provide a large thermal mass because the reaction was temperature-sensitive. The microplate was immediately placed at 50°C for 180 min, and the absorbance was monitored at 470 nm every 30 min. Results, from three independent assays, were expressed as percentage of antioxidant activity (% AA) measured on the basis of BCB inhibition and calculated as follows:

(1) $$\begin{array}{c} \%\;AA=\left(1-\frac{Abs\;{sample}_{T_{0^{\prime }}}-Abs\;{sample}_{T_{180^{\prime }}}}{Abs\;{blank}_{T_{0^{\prime }}}-Abs\;{blank}_{T_{180^{\prime }}}}\right)*100. \end{array}$$

#### 2.7.4. RACI Calculation

To get a complete and dynamic picture of the ranking of food antioxidant capacity, the relative antioxidant capacity index was calculated. Previous data confirmed that RACI is a valid tool to assess food antioxidant capacity [30]. RACI was calculated by integrating the antioxidant capacity values generated from the different cell-free model methods.

### 2.8. *In Vitro* Activity

#### 2.8.1. Cell Culture

H9c2 cells (normal primary cardiomyocytes; ATTC, Manassas, VA, USA) were cultured, following the usual procedures, in DMEM supplemented with 10% FBS, 100 units/mL penicillin, and 100 mg/mL streptomycin in tissue culture flasks at 37°C in a humidified atmosphere of 5% CO_2_.

#### 2.8.2. Cell Viability

H9c2 cells were seeded at a density of 10 × 10^3^ cells/cm^2^ in 96-well plates. “Fantastico” bergamot juice was dissolved in DMSO at a concentration of 100 mg/mL (stock preparation). After 24 h, the cells were treated for three days with fresh growth medium containing the lyophilized “fantastico” bergamot juice dissolved in DMSO, ranging from 0.01 to 1 mg/mL (DMSO 0.01%), or vehicle. Cell viability was then determined using the MTS assay according to manufacturer's instruction. The absorbance of formazan at 490 nm was measured in a colorimetric assay with an automated plate reader from three independent assays (Victor Wallac 2, Perkin Elmer).

#### 2.8.3. Senescence-Associated *β*-Galactosidase Staining

Cell senescence in H9c2 cells was induced by DOX or H_2_O_2_, as previously reported [31, 32]. Briefly, cells were cultured up to about 80% confluence in DMEM medium; before the experiments, cells were seeded onto 24-well plates at a density of 10 × 10^3^ cells/cm^2^. After time to allow the cell attachment (24 h), the medium was replaced in each well and the cells received different treatments for 3 h, H_2_O_2_ (ranging from 5 to 100 *μ*M), DOX (0.01, 0.05, and 0.1 *μ*M), or relative vehicles. Subsequently, the cells were cultured for 3 days, after which senescence was determined.

To evaluate the number of senescent cells after 3 days from DOX or H_2_O_2_ insults, the senescence marker sa-*β*-Gal was detected as previously reported [33]. In parallel experiments, different concentrations of lyophilized “fantastico” bergamot juice dissolved in DMSO (0.01, 0.1 and 1 mg/mL) or vehicle (0.01% DMSO) were added to the cell medium after the DOX or H_2_O_2_ senescence insults and maintained in the medium for three days before assessing the sa-*β*-Gal staining. According to the literature, treated cells were fixed in *p*-formaldehyde and incubated in a dry incubator in freshly prepared staining solution for 16 h at 37°C [34]. Cells were then washed in phosphate-buffered saline (PBS) (1x) and photographed at 100x magnification. Images of random light microscopic fields were captured (5 fields per well), and both blue and total cells were counted using ImageJ (ImageJ Software, version 1.41, USA). Three independent assays were performed.

### 2.9. *In Vivo* Activity

#### 2.9.1. Chronic Treatment

C57BL/6J mice were taken from Envigo (Milan, Italy). All procedures were performed according to European (EEC Directive 2010/63) and Italian (D.L. 4 March 2014 n. 26) legislation. Animals were housed in cages with free access to standard food pellets and water on a 12 h light/dark cycle.

Nine-month-old male mice (25–30 g) were randomly assigned into two groups: one group was used as a control while the other group received lyophilized “fantastico” bergamot juice diluted in water (1 mg/kg/day). The final concentration of the lyophilized juice was 1 mg/mL, and the diluted solution was daily prepared. Mice were weekly weighed, and water intake was daily monitored over a period of three months. At the end of treatment, mice were fasted overnight to measure blood glucose levels. Blood was collected from the tail tip, and glucose concentration was determined using the Glucocard™ blood glucose meter (Menarini). Then, mice were anaesthetized with an intraperitoneal injection of aqueous urethane solution 30% *w*/*w* (Sigma-Aldrich). Intracardiac blood was collected in tubes with the anticoagulant EDTA (BD Vacutainer), in order to measure the complete lipid panel (tryglicerides, total cholesterol, HDL, and LDL) and glycated haemoglobin (using cobas b 101 instrument, Roche Diagnostics). Finally, hearts were taken, and an amount was immediately processed to extract the total RNA, in order to assess three genes relevant to aging, lifespan, and antioxidant response (SIRT1, NRF2, FOXO3, HO-1, and NQO1).

#### 2.9.2. RNA Extraction and Real-Time PCR Analysis

Total RNA from mouse hearts was extracted using the RNeasy® Mini Kit (Qiagen, Hilden, Germany) according to the manufacturer's instructions. Purity of the RNA samples was determined by measuring the absorbance at 260 : 280 nm. cDNA synthesis was performed with 500 ng of RNA using the i-Script cDNA synthesis kit (Bio-Rad, Hercules, USA) following the manufacturer's instructions. Primers used for RT-PCR were designed in intron/exon boundaries to ensure that products did not include genomic DNA (Table 1) [35–37]. RT-PCR reactions consisted of 25 *μ*L Fluocycle® II SYBR® (EuroClone, Milan, Italy), 1.5 *μ*L of both 10 *μ*M forward and reverse primers for SIRT1, NRF2, FOXO3, HO-1, NQO1, and GAPDH (Sigma-Aldrich, Milan, Italy), 3 *μ*L cDNA, and 19 *μ*L of H_2_O. All reactions were performed for 38 cycles using the following temperature profiles: 94°C for 30 s (initial denaturation); Annealing temperature °C (see Table 1) for 30 s (annealing); and 72°C for 1 s (extension). GAPDH was used as the housekeeping gene. mRNA levels for each sample were normalized against GAPDH mRNA levels, and relative expression was calculated by using the Ct value. PCR specificity was determined by both the melting curve analysis and gel electrophoresis.

### 2.10. Statistical Analyses

The nonlinear multipurpose curve-fitting program GraphPad Prism (GraphPad Software Inc., San Diego, CA) was used for data analysis and graphic presentations. The cell-free model antioxidant activity data were expressed as mean ± standard deviation of three independent experiments. Pearson coefficient was used to determine the correlation among polyphenol and flavonoid contents and antioxidant activity. The *in vitro* antisenescence activity data are presented as the means ± standard errors of the means (SEM) of triplicate samples and are representative of three different experiments. Statistical analysis was performed by one-way analysis of variance (ANOVA) with Bonferroni's corrected *t*-test for post hoc pair-wise comparisons. *p* < 0.05 was considered statistically significant.

## 3. Results and Discussion

### 3.1. Juice Composition

**Figure 1:**
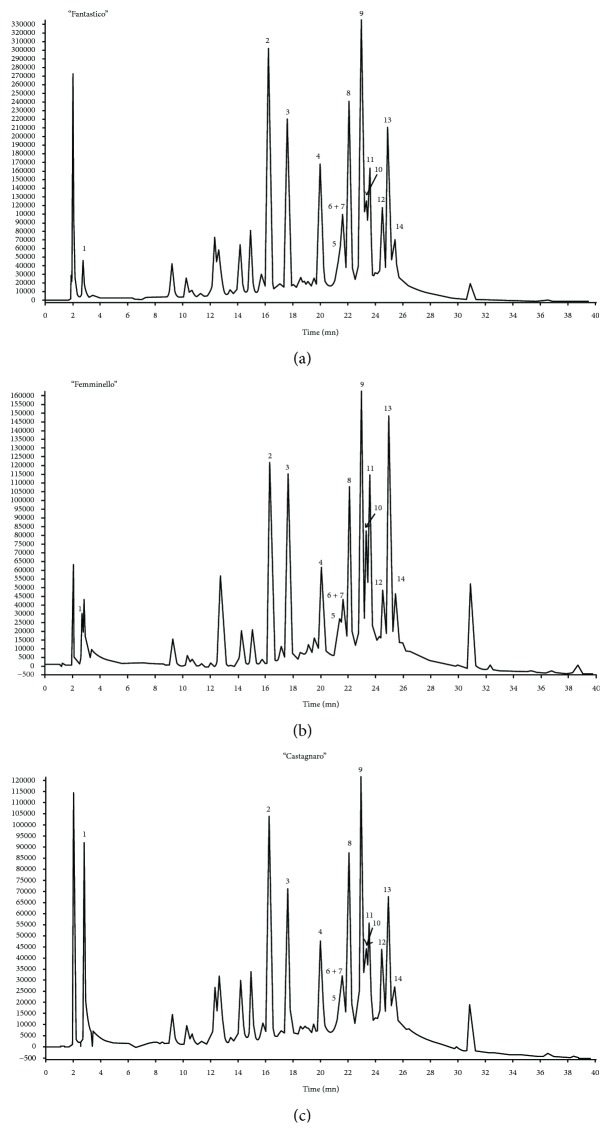
HPLC-PDA profiles of *C. bergamia* juices from “fantastico” (a), “femminello” (b), and “castagnaro” (c) cultivars. Peaks were monitored at 325 nm. For the peak data, see Table 2.

In the present study, the HPLC-PDA profiles recorded at 325 nm of the three *C. bergamia* juices obtained from the different cultivars are displayed in Figure 1.

Compounds **1**–**14** were detected in all cultivars and were identified comparing their HPLC elution orders, ESI-MS/MS, and UV data (Table 2) with those previously reported.

In agreement with previous studies, our investigation about the chemical composition of bergamot juices led to identification of citric acid (**1**) [38], the flavonoids vicenin-2 (**2**), lucenin-2 4′-methyl ether (**3**), neoeriocitrin (**4**), naringin (**8**), neohesperidin (**9**), 3-hydroxy-3*-*methylglutaryl-neoeriocitrin (**10**), neodiosmin/chrysoeriol 7-O-neohesperidoside (**11**), melitidin (**12**), and brutieridin (**13**) [39, 40]. The ESI-MS/MS technique has been useful for the identification of components that can occur as isomers in the *Citrus* juice. In particular, compound **4** was distinguished by its isomer eriocitrin due to its fragmentation pattern in which the base peak is represented by product ion at *m*/*z* 459 instead of *m*/*z* 287, as observed in the MS/MS experiment of eriocitrin [39]. Similarly, the base peak generated by MS/MS analysis of compound **8** was observed at *m*/*z* 459, in agreement with the fragmentation pathway of naringin [39], whereas the isomer narirutin is characterized by a most abundant product ion at *m*/*z* 287 [41]. The obtained data confirmed the bergamot juice composition, well established in many previous studies demonstrating that it is rich in flavonoid constituents, including both flavone and flavanone glycosides [39].

In addition, herein, the two limonoids, limonin glucoside (**5**) and nomilinic acid glucoside (**14**), were also identified. Limonoids are a class of bitter components of bergamot fruits and are generally abundant in *Citrus* seeds, but they are also present in pulps, peels, and juices, as reported in a recent work [40].

**Figure 2:**
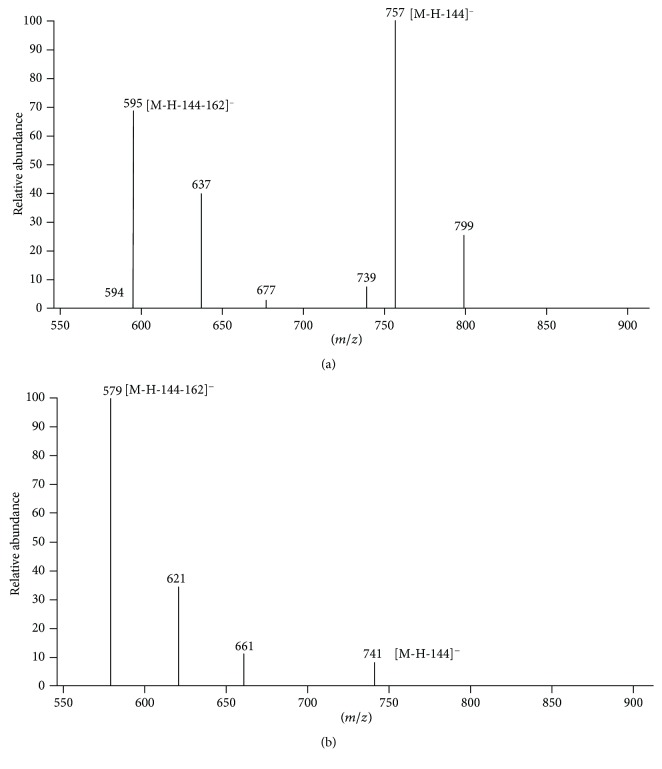
ESI-MS/MS spectra of compounds **6** and **7**. ESI-MS/MS spectra of compound **6** at *m*/*z* 901 (a) and compound **7** at *m*/*z* 885 (b), performed in the negative ion mode. For the peak data, see Table 1.

Lastly, the peak at retention time (*t*_R_) 21.62 min was generated by the coelution of two compounds (**6** and **7**) that have not been previously detected in *C. bergamia*. Compounds **6** and **7** remained not identified, but we tentatively hypothesized their structures basing on full MS and MS/MS spectra (Figure 2).

Full spectrum of compound **6** displayed a deprotonated molecule [M−H]^−^ at *m*/*z* 901. The MS/MS product ion spectrum showed ions at *m*/*z* 757 generated by the loss of a 3-hydroxy-3-methylglutaryl moiety ([M−H−144]^−^) and 595, resulting from the loss of a 3-hydroxy-3-methylglutaryl residue followed by one hexose molecule ([M−H−144−162]^−^). The base peak ion at *m*/z 595 can be attributed to eriocitrin or neoeriocitrin, two flavanone O-glycoside isomers previously reported in *C. bergamia* juice [39]. A very similar fragmentation profile was shown by MS/MS of component **7**, obtained at *m*/*z* 855, producing ions at *m*/*z* 741 ([M−H−144]^−^) and 579 ([M−H−144−162]^−^), leading to suppose that compound **7** can be a naringin or narirutin ([M−H]^−^ at *m*/*z* 579) with a hydroxy-3-methylglutaryl moiety and a hexose residue linked to the molecule.

The quantification of the main flavonoids detected in all sample juices was determined on the naringin basis by HPLC-PDA recorded at 325 nm. Results obtained from quantitative analysis are listed in Table 3.

### 3.2. Total Polyphenol and Flavonoid Content

TPC and TFC of juices obtained from “fantastico,” “femminello,” and “castagnaro” cultivars of *C. bergamia* fruits were then investigated. Each cultivar showed different phenolic and flavonoid contents (Table 4).

In particular, TPC ranged from 8.77 ± 0.77 to 17.10 ± 1.34 mg GAE/g of the sample in “castagnaro” and “fantastico,” respectively. TFC, instead, varied from 6.51 ± 0.61 to 57.46 ± 3.20 mg QE/g of the sample again in “castagnaro” and “fantastico,” respectively. The values of TPC are in accordance with Sicari and Pellicanò [42], although in that case, fresh juice has been used as the sample, whereas here juices have been previously lyophilized.

### 3.3. In Tube Antioxidant Activity

In order to characterize the total antioxidant capacity of bergamot juices [27], four different and complementary assays were herein used to evaluate juice antioxidant activities. The samples were evaluated firstly for their antiradical activity against superoxide and nitric oxide physiological radicals. Then, samples were assessed for their abilities to reduce ferric ions and to inhibit the lipid peroxidation (Table 4). Two of the used tests (O_2_^•−^ and FRAP) have not been previously reported for measuring the activity of bergamot juice samples. Last, the RACI was also calculated.

#### 3.3.1. O_2_^•−^ Scavenging Activity

O_2_^•−^ is a radical oxygen species normally produced inside the body, but it is known to be very harmful to cellular components as a precursor of a more reactive oxygen species, for example, the hydroxyl radical. The activity of ascorbic acid (IC_50_ = 4.34 ± 0.39 mg/mL), used as reference, was compared with the samples by IC_50_ values (Table 4). Juice obtained from “castagnaro” exhibited the lowest IC_50_ value (IC_50_ = 1.01 ± 0.02 mg/mL), followed by “fantastico” (IC_50_ = 1.13 ± 0.11 mg/mL). Both these juices demonstrated to have a stronger superoxide radical scavenging activity than ascorbic acid. Juice obtained from “femminello” exhibited an activity (IC_50_ = 4.77 ± 0.58) similar to ascorbic acid. The scavenging activities were in accordance with previous investigation [10].

#### 3.3.2. Ferric Reducing Antioxidant Power

FRAP test evaluates the ability of plant extracts to reduce ferric ions. In our study, the FRAP assay revealed that “fantastico” cultivar had the highest reducing power (9.83 ± 0.54 mg TE/g), followed by “femminello” and “castagnaro” (8.43 ± 0.67 and 6.85 ± 0.63 mg TE/g, respectively) (Table 4).

#### 3.3.3. Lipid Peroxidation Inhibition Test

To get a wider overview of the antioxidant potential, the antioxidant effect of the samples on the peroxidation of linoleic acid in the *β*-carotene/linoleic acid system was also investigated by a BCB test. The oxidation of linoleic acid generates peroxyl free radicals, which will then oxidize the highly unsaturated *β*-carotene. The presence of antioxidants minimizes the oxidation of *β*-carotene. All samples showed moderate *β*-carotene bleaching inhibition activity; in fact, results ranged from 22.42 ± 1.15 to 31.60 ± 0.50 % and the highest value was found in cultivar “castagnaro” (Table 4).

#### 3.3.4. Relative Antioxidant Capacity Index

**Figure 3:**
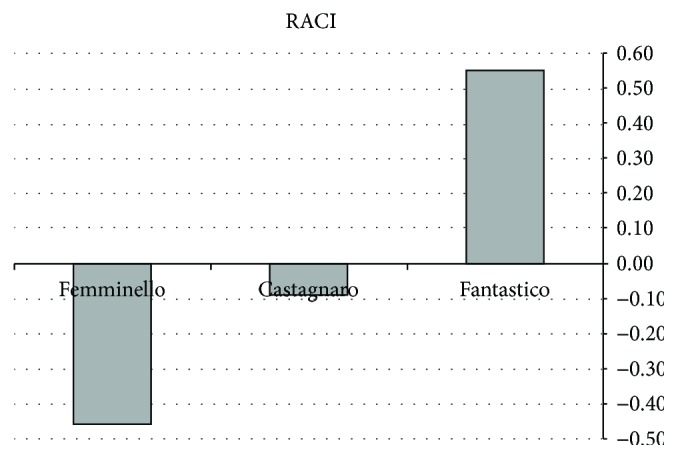
RACI values obtained for the *C. bergamia* cultivars. RACI values were obtained comparing the total phenolic content, the superoxide radical scavenging activity, the ferric reducing antioxidant power, and the lipid peroxidation inhibition results of investigated cultivars.

Moreover, RACI was calculated among all the tested samples. All methods used for antioxidant activity determination together with TPC were included in RACI calculation. In particular, TPC assay results were included since it was recently proposed that the results obtained by the Folin-Ciocalteu procedure could be also interpreted as an alternative way to measure the total reducing capacity of samples as the reagent reacts with any reducing substance [26]. Results of antioxidant activity expressed as IC_50_ were converted in 1/IC_50_ before the RACI calculation, and data of relative antioxidant activity were represented as histograms (Figure 3).

According to the obtained results, “fantastico” cultivar had the highest RACI (0.55), followed by “castagnaro” (−0.09), and “femminello” (−0.45).

Recently, Sicari and Pellicanò [42] have investigated *C. bergamia* cultivar juices using two radical scavenging assays (2,2′-azino-bis-3-ethylbenzthiazoline-6-sulphonic acid, ABTS, and 1,1-diphenyl-2-picrylhydrazyl, DPPH). The highest antioxidant capacity has been found in “castagnaro” juice compared to “fantastico” and then “femminello”. On the contrary, comparing the radical scavenging activity of the three bergamot cultivars using just DPPH assay, the previous paper [39] has established “femminello” more active than “fantastico” and “castagnaro” cultivars.

These discrepancies among literature and in respect to present results may be ascribed to the fruit phenolic content that depends on many factors, such as the degree of maturity at harvest, genetic differences, and environmental conditions [42]. Notably, statistically significant differences on antioxidant activity and phenolic composition of bergamot fruits collected in different Calabria areas have been evidenced, suggesting that also the microclimate may influence the chemical composition and, therefore, the quality of the juice itself [11].

Then, in order to verify if there was a correlation between juice amount of polyphenols and antioxidant properties, Pearson analyses were conducted using averaged values of each variable, as reported in Table 5.

The highest positive correlation was observed between FRAP and TPC and TFC (*r* = 0.89), suggesting a consistent relationship between polyphenolic and flavonoid content and the ferric reducing antioxidant power.

On the other hand, it was not noticed a high value of correlation when O_2_^•−^ and BCB were evaluated in comparison to phenolic and flavonoids content. This result could be possibly explained because these tests involved not only phenolic compounds [27]. Moreover, the BCB test mostly gives an indication of lipophilic active constituents, whereas TPC assay reports the levels of both lipophilic and hydrophilic phenols. These data could suggest the presence of minor lipophilic and/or not phenolic compounds acting synergistically to enhance the biological activity.

### 3.4. Antisenescence Activity

#### 3.4.1. H9c2 Mitochondrial Metabolic Activity

Although from embryonal origin, H9c2 cells were found to be closer for energy metabolism features to normal primary cardiomyocytes, H9c2 cells have been successfully used as an in vitro model to simulate cardiac ischemia-reperfusion injury [43]. Moreover, this cell line is widely used as a successful cellular model for studies of myocardial pathophysiology including aging processes [31, 32].

**Figure 4:**
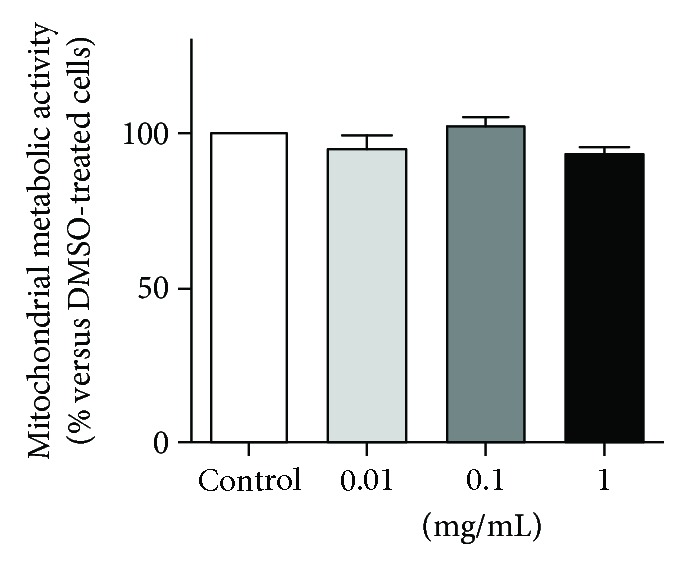
Effects of *C. bergamia* “fantastico” cultivar juice on the mitochondrial oxidative metabolism activity. H9c2 cells were treated with three different concentrations of lyophilized juice, dissolved in DMSO, and the mitochondrial oxidative metabolism activity was examined.

As a first step, in order to assess if bergamot juice affected per se cellular life/death processes, the mitochondrial metabolic activity was explored in H9c2 cells by the use of a tetrazolium dye. The mitochondrial metabolic activity is a parameter conventionally used to estimate cellular proliferation and drug toxicity. Cells were treated with the lyophilized bergamot juice of “fantastico” cultivar, which showed the highest antioxidant potential and represent about 90% among the three cultivars. The results demonstrated that bergamot juice did not exert any cytotoxic effect on H9c2 cells (Figure 4). Indeed, no difference was observed in the mitochondrial metabolic activity following three days of treatment in samples compared to control.

#### 3.4.2. H9c2 Cell Senescence Induction

H9c2 cell senescence was induced by cell exposure to exogenous oxidative insults (doxorubicin, DOX, or hydrogen peroxide, H_2_O_2_), as previously reported [31, 32, 34]. The presence of senescence-associated *β*-galactosidase (sa-*β*-Gal) was monitored as a marker of cellular senescence. For chemical insults, in order to establish the effective dose of DOX or H_2_O_2_ able to induce cellular senescence, H9c2 cells were challenged with increasing concentrations of DOX (ranging from 0.01 to 0.1 *μ*M) or H_2_O_2_ (ranging from 5 to 100 *μ*M), diluted in water, for 3 h. Subsequently, the cells were cultured with fresh complete medium for 3 days, and then the presence of sa-*β*-Gal was examined. A concentration-dependent increase of sa-*β*-gal was observed following the H9c2 cell treatment with DOX (0.05 *μ*M, *p* < 0.01 and 0.1 *μ*M, *p* < 0.001). Conversely, only 60 *μ*M H_2_O_2_ was effective to induce the staining of sa-*β*-Gal significantly (*p* < 0.01). A higher dose of H_2_O_2_ (100 *μ*M) did not further increase the senescence marker, probably due to the cytotoxic effect exerted by such a high concentration of H_2_O_2_. Together, these results allowed us to establish 0.05 *μ*M DOX and 60 *μ*M H_2_O_2_ as the effective drug concentrations to be used for the following H9c2 cell experiments.

#### 3.4.3. Effects of Bergamot Juice in Cellular Senescence Models

**Figure 5:**
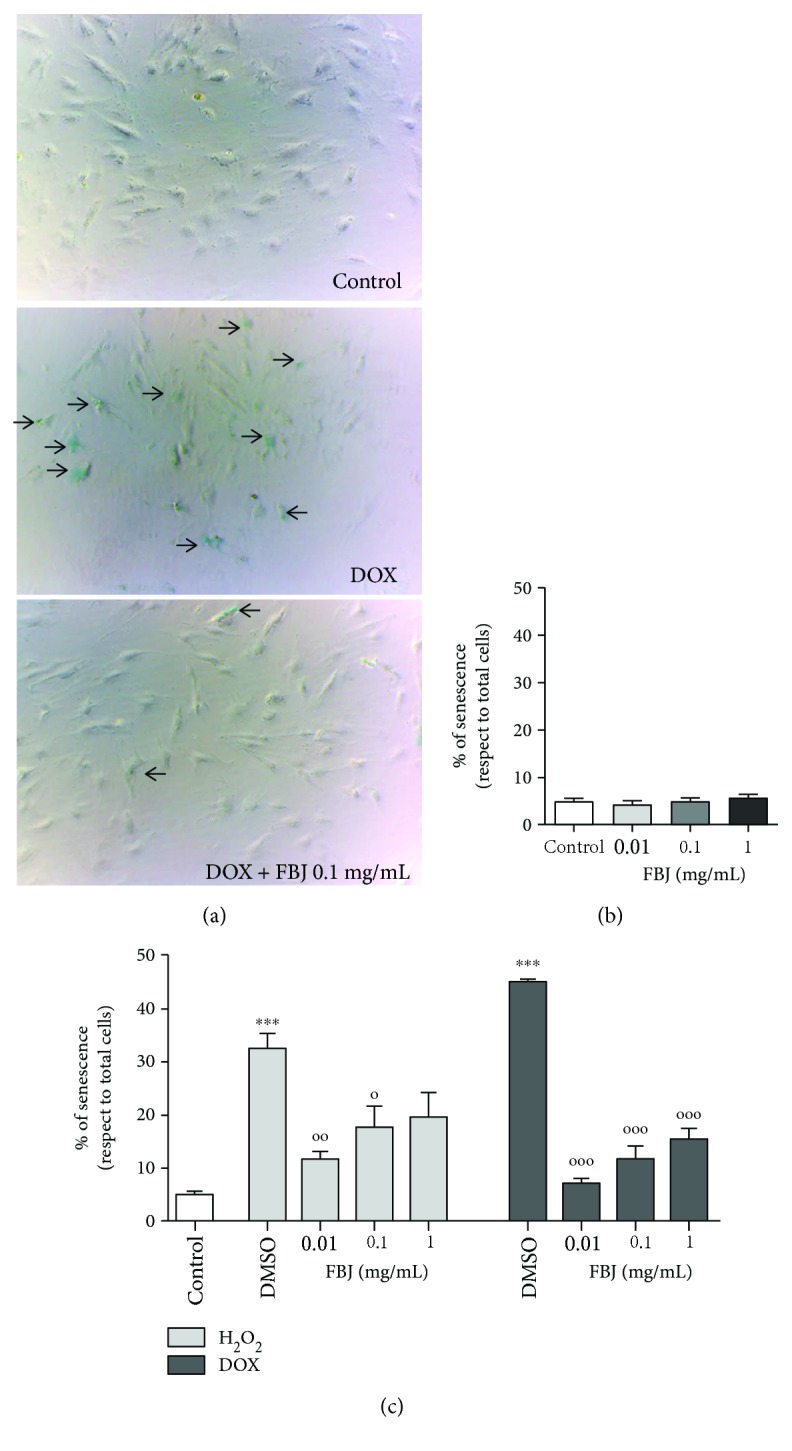
Effects of *C. bergamia* “fantastico” juice on H9c2 senescence-associated *β*-galactosidase staining. (a) Representative phase contrast photomicrographs of control cells, DOX-injured cells, and DOX-injured cells treated with FBJ, 0.1 mg/mL. The arrows indicate the blue-stained cells. (b) Percentage of cellular senescence in not-injured cells treated with different concentrations of FBJ. Data are shown as the percentages of *β*-galactosidase-positive cells with respect to the total cell number of the sample. Each bar represents the mean ± SEM of three replicates from three independent experiments. (c) Percentage of cellular senescence in H_2_O_2_- or DOX-injured cells treated with FBJ at different concentrations. Data are shown as the percentages of *β*-galactosidase-positive cells with respect to the total cell number of the sample. Each bar represents the mean ± SEM of three replicates from three independent experiments. The light gray bars represent the data obtained from the H_2_O_2_-induced senescence model; the dark gray bars represent the data obtained from the DOX-induced senescence model. ^∗∗∗^*p* < 0.001 versus the control (cells not injured); °°°*p* < 0.001 versus the H_2_O_2_- or DOX-challenged cells with DMSO; °°*p* < 0.01 versus the H_2_O_2_- or DOX-challenged cells with DMSO; °*p* < 0.05 versus the H_2_O_2_- or DOX-challenged cells with DMSO.

Therefore, with the aim to explore the protective potential of bergamot juice against cellular senescence, the DOX- or H_2_O_2_-treated cells were cultured with fresh complete medium containing different concentrations of “fantastico” bergamot juice (FBJ) and the pivotal senescence hallmark, the sa-*β*-Gal staining, was assessed after 3 days.  Figure 5 showed the results obtained from the H_2_O_2_-induced (light gray bars) and DOX-induced (dark gray bars) senescence model.

In Figure 5(a), representative phase contrast photomicrographs were reported, in which the sa-*β*-Gal staining cells are in light blue. As reported in Figure 5(b), bergamot juice alone did not affect the number of senescent cells with respect to control, for any of the tested concentrations. As expected, H9c2 cells injured with H_2_O_2_ or DOX evidenced a significant sa-*β*-Gal staining in respect of control cells (*p* < 0.001, ∗ versus control cells; Figure 5(c)), and notably, all the tested “fantastico” bergamot juice cotreatments were able to protect the injured cells from the appearing of the senescence hallmark (*p* < 0.05 and *p* < 0.01, ° versus H_2_O_2_ or DOX-treated cells; Figure 5(c)). Interestingly, in both cellular models, the relation between senescence percentages and FBJ concentrations showed an inversely proportional trend. These data suggested that the optimal FBJ concentration capable of better counteracting the senescence mechanism is the lowest one (0.01 mg/mL).

To the best of our knowledge, there is another study in the literature concerning the antisenescence potential of bergamot; this study has evidenced antiaging and immune modulating responses on human photoaged keratinocytes treated with a highly concentrated extract of bergamot fruit [44]. In addition, our results are in accordance with previous findings suggesting that the bergamot juice polyphenols could influence cellular function by acting as activators of sirtuin-1 [9], a nuclear histone deacetylase, largely involved in aging processes [45].

#### 3.4.4. Effects of Bergamot Juice In Vivo

In light of the antisenescence results, “fantastico” juice effects were also assessed in old mice. During daily water intake of bergamot fruit juice, no difference between the “bergamot group” and the “vehicle group” was highlighted along the three months of treatment. Weekly weightings showed a physiological increase in the body mass, according to growth curves reported in the literature (data not shown). Blood total cholesterol, HDL, LDL, triglyceride, glycaemia, and glycated haemoglobin values were superimposable with the corresponding values measured in young-adult animals, suggesting that these animals did not have cardiometabolic diseases. Indeed, lipid and glycemic parameters measured in BFJ and control groups were not significantly different (Table 6).

**Figure 6:**
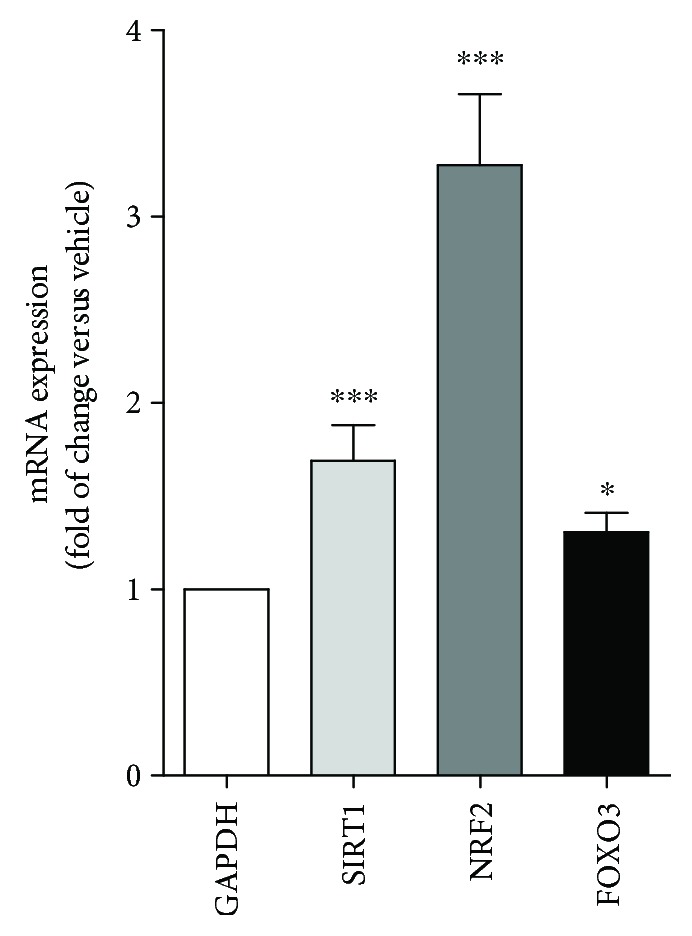
FBJ induces gene expression. Real-time PCR analyses showed a statistically significant increase in SIRT1, NRF2, and FOXO3 mRNA levels in hearts of old mice fed with FBJ for three months. ^∗^*p* < 0.05; ^∗∗∗^*p* < 0.005.

Nevertheless, the real-time PCR analyses, performed on hearts of the old mice fed with FBJ or vehicle for three months, showed statistically significant increases in mRNA levels of three regulator genes, SIRT1, NRF2, and FOXO3, which are largely involved in antiaging and antioxidant responses (Figure 6).

The silent information regulator 2 family proteins (SIRT), also called “sirtuins,” have been demonstrated to coordinate metabolic responses to changes in nutritional availability and maintain physiological homeostasis. Brain-specific Sirt1-overexpressing (BRASTO) transgenic mice have shown significant life span extension and delay in the aging process compared to control mice [46]. The nuclear factor erythroid 2-related factor 2 (NRF2) is a regulator of cellular resistance to oxidants; it orchestrates the expression of antioxidant response element-dependent genes to regulate the outcomes of oxidant exposure [47]. Last, forkhead/winged helix box gene, group O (FOXO) proteins are a set of transcription factors at a central integration hub for many important cellular pathways, relevant to healthy aging and longevity. FOXOs are involved in energy metabolism, oxidative stress, proteostasis, apoptosis, cell cycle regulation, metabolic processes, immunity, inflammation, and stem cell maintenance [48]. Notably, a BFJ-enriched diet was able to increase the mRNA expression of these three key genes in mice hearts, suggesting an enhanced cellular antioxidant capacity and an antiaging response for those mice fed with BFJ.

**Figure 7:**
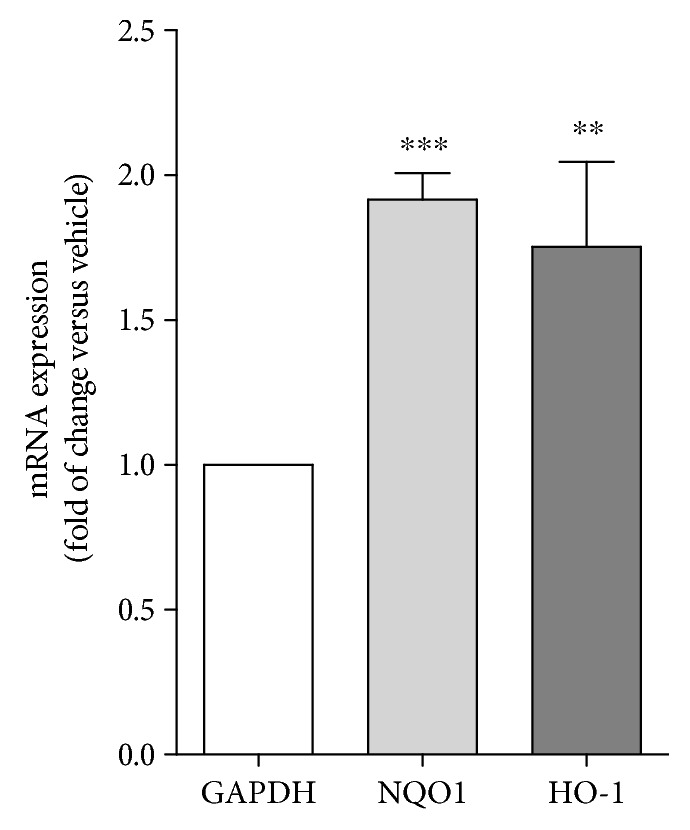
FBJ induces NQO1 and HO-1 gene expression. Real-time PCR analyses showed a statistically significant increase in NQO1 and HO-1 mRNA levels in hearts of old mice fed with FBJ for three months. ^∗∗^*p* < 0.01; ^∗∗∗^*p* < 0.005.

In order to further demonstrate the involvement of these pathways in the antioxidant effects of the active compounds contained in bergamot juice, two additional genes, NAD(P)H dehydrogenase quinine 1 (NQO1) and heme oxygenase 1 (HO-1), routinely assessed as markers of NRF2 activity, have been investigated. These two NRF2 target genes are markers of a robust antioxidant response [49]. As reported in Figure 7, the real-time PCR analyses, performed on hearts of the old mice fed with FBJ or vehicle for three months, showed statistically significant increases in mRNA levels of NQO1 and HO-1, suggesting a robust antioxidant effect for FBJ (Figure 7).

Then, considering that oxidative stress has been pointed out as relevant cause of both in heart aging and in the development of several cardiac diseases, an approach with BFJ could guarantee an improvement of these targets (SIRT1, NRF2, FOXO, HO-1, and NQO1) typically altered and at the basis of cardiac dysfunction and of the consequent tissue damage [50].

Therefore, all together, these data indicated that the bergamot juice exerts beneficial health effects counteracting senescence in myocardial H9c2 cells and increases the antiaging and antioxidant defenses in mouse hearts, opening new perspectives on the potential pharmaceutical application of this natural source.

## 4. Conclusions

Natural polyphenols have gained considerable attention as potential agents for prevention and treatment of oxidative stress-related diseases, such as aging and cardiovascular diseases [5, 51], and in particular, the Mediterranean citrus fruit bergamot has been demonstrated to be a valuable source of antioxidant molecules. In addition, limonoids occurring in *Citrus* genus displayed different pharmacological activities; in particular, a placebo-controlled, double-blind study demonstrated that limonin glucoside may be useful in the prevention and/or treatment of different chronic inflammatory diseases, such as cancer, diabetes, and cardiovascular diseases [52]. The results of our study confirmed that bergamot juices possess a robust antioxidant activity and could be considered a valuable source of health-promoting constituents, such as naringin, neoeriocitrin, and neohesperidin, as previously established [11, 39, 41]. Furthermore, to the best of our knowledge, we demonstrated for the first time the potential of bergamot juice to counteract also the senescence processes, *in vitro* and *in vivo*.

These findings supported the nutraceutical properties of the bergamot fruit, encouraging the use of its pulp as health-promoting resource.

## Figures and Tables

**Table 1:** Nucleotide sequences, annealing temperature, and product size of the primers utilized in real-time RT-PCR experiments.

		Primer nucleotide sequences	Annealing temp (°C)	Product size (base pairs)
SIRT1	For. 5	ATGACGCTGTGGCAGATTGTT	66.8	202
	Rev. 5′	CCGCAAGGCGAGCATAGAT	67.4	

NRF2	For. 5′	GGACATGGAGCAAGTTTGGC	66.8	165
	Rev. 5′	TCCAGCGAGGAGATCGATGA	68.4	

FOXO3	For. 5′	AGTGGATGGTGCGCTGTGT	67.1	100
	Rev. 5′	CTGTGCAGGGACAGGTTGT	64.0	

HO-1	For. 5′	ATACCCGCTACCTGGGTGAC	64.9	200
	Rev. 5′	TGTCACCCTGTGCTTTGACCT	67.0	

NQO1	For. 5′	TTCTGTGGCTTCCAGGTCTT	63.8	130
	Rev. 5′	AGGCTGCTTGGAGCAAAATA	63.7	

GAPDH	For. 5′	ATGTGTCCGTCGTGGATCTGAC	68.5	132
	Rev. 5′	AGACAACCTGGTCCTCAGTGTAG	63.4	

**Table 2:** Spectral (UV and ESI-MS/MS), chromatographic data of compounds **1**–**14**, detected in *C. bergamia* juices from “fantastico,” “femminello,” and “castagnaro” cultivars.^a^

Peak	Compound	*t*_R_ (min)	[M−H]^−^	MS/MS base peak	MS/MS ions (*m*/*z*)	*λ*_max_ (nm)
Organic acids						
**1**	Citric acid	2.74	191	111	129, 173, 87	210

Flavone C-glucoside						
**2**	Vicenin-2	16.22	593	473	575, 503, 383, 353	270, 335
**3**	Lucenin-2 4′-methyl ether	17.57	623	503	605, 533, 503, 413, 383	255, 270, 345

Flavanone O-glycosides						
**4**	Neoeriocitrin	19.98	595	459	441, 287, 235, 205	285
**8**	Naringin	22.06	579	459	417, 313, 271	280, 330
**9**	Neohesperidin	22.97	609	301	489, 447	285, 330

Flavone O-glycosides						
**10**	3-Hydroxy*-*3*-*methylglutaryl-neoeriotricin	23.19	739	595	637, 677, 459	285, 325
**11**	Neodiosmin/chrysoeriol 7-O-neohesperidoside	23.57	607			250, 280, 330

3-Hydroxymethylglutaryl flavanone O-glycosides						
**6**	Unidentified	21.62	901	757	839, 799, 677, 637, 595	270, 345
**7**	Unidentified	21.62	885	579	741, 661, 621	265, 345
**12**	Melitidin	24.47	723	579	661, 621, 579	285, 325
**13**	Brutieridin	24.92	753	609	691, 651, 301	285, 325

Limonoid glucosides						
**5**	Limonin glucoside	21.41	649	605	461, 443	210
**14**	Nomilinic acid glucoside	25.40	711	607	651	210

^a^Compound numbers correspond with peak numbers in Figure 1.

**Table 3:** Quantitative amount (mg/L of fresh bergamot juice) of the main flavonoids detected in *C. bergamia* juices from “fantastico,” “femminello,” and “castagnaro” cultivars by HPLC-PDA analysis at 325 nm.^a^

Peaks	Compounds	Cultivars		
		“Fantastico”	“Femminello”	“Castagnaro”
**2**	Vicenin-2	187.4	78.3	63.0
**3**	Lucenin-2 4′-methyl ether	140.8	74.4	43.8
**4**	Neoeriocitrin	103.7	78.3	29.3
**8**	Naringin	112.8	51.2	42.6
**9**	Neohesperidin	157.2	91.4	58.0
**12**	Melitidin	11.2	16.1	16.0
**13**	Brutieridin	94.1	66.1	27.4

^a^Compound numbers correspond with peak numbers in Figure 1.

**Table 4:** Results of O_2_^•−^, FRAP, and BCB tests, beside TPC and TFC, of juices of *C. bergamia* fruits obtained from cultivars “fantastico,” “femminello,” and “castagnaro”. mg GAE/g = mg of gallic acid equivalents per gram of the dried sample; mg QE/g = mg of quercetin equivalents per gram of the dried sample; IC_50_ = concentration of the sample required to inhibit the activity by 50% in mg/mL; mg TE/g = mg of Trolox equivalents per gram of the dried sample; % AA = percentage of antioxidant activity at final concentration of 0.25 mg/mL.

Cultivars	TPC (mg GAE/g)	TFC (mg QE/g)	O_2_^•−^ (IC_50_ mg/mL)	FRAP (mg TE/g)	BCB (% AA)
“Fantastico”	17.10 ± 1.34	57.46 ± 3.20	1.13 ± 0.11	9.83 ± 0.54	26.18 ± 0.85
“Femminello”	14.00 ± 0.56	16.89 ± 1.50	4.77 ± 0.58	8.43 ± 0.67	22.42 ± 1.15
“Castagnaro”	8.77 ± 0.77	6.51 ± 0.61	1.01 ± 0.02	6.85 ± 0.63	31.60 ± 0.50
Ascorbic acid	—	—	4.34 ± 0.39	—	—

**Table 5:** Pearson coefficient calculated among TPC, TFC, superoxide radical scavenging activity, ferric reducing antioxidant power, and lipid peroxidation inhibition.^a^

	TPC	TFC
O_2_^•−^	−0.27	0.20
FRAP	**0.99**	**0.93**
BCB	−0.70	−0.29

^a^In bold face the highest positive correlations.

**Table 6:** Lipid panel, glycaemia, and glycated haemoglobin levels (HbA1c) of treated mice.

	Vehicle	FBJ (1 mg/mL)
Total cholesterol (mg/dL)	69.5 ± 0.5	68 ± 2.57
HDL (mg/dL)	19 ± 4	17.8 ± 1.74
LDL (mg/dL)	38.5 ± 5.5	37.48 ± 3.06
Triglycerides (mg/dL)	57 ± 2	62 ± 1.9
Glycaemia (mg/dL)	91.5 ± 10.5	85.4 ± 9.5
HbA1c (mmol/mol)	29 ± 3	27.4 ± 0.87

## Data Availability

The data used to support the findings of this study are all included and available within the article.
